# Clinical Pathology, Immunopathology and Advanced Vaccine Technology in Bovine Theileriosis: A Review

**DOI:** 10.3390/pathogens9090697

**Published:** 2020-08-25

**Authors:** Onyinyechukwu Ada Agina, Mohd Rosly Shaari, Nur Mahiza Md Isa, Mokrish Ajat, Mohd Zamri-Saad, Hazilawati Hamzah

**Affiliations:** 1 Department of Veterinary Pathology and Microbiology, Faculty of Veterinary Medicine, Universiti Putra Malaysia, Serdang 43400, Malaysia; nurmahiza@upm.edu.my; 2 Department of Veterinary Pathology and Microbiology, Faculty of Veterinary Medicine, University of Nigeria Nsukka, Nsukka 410001, Nigeria; 3 Animal Science Research Centre, Malaysian Agricultural Research and Development Institute, Headquarters, Serdang 43400, Malaysia; rosly@mardi.gov.my; 4 Department of Veterinary Pre-clinical sciences, Faculty of Veterinary Medicine, Universiti Putra Malaysia, Serdang 43400, Malaysia; mokrish@upm.edu.my; 5 Research Centre for Ruminant Diseases, Faculty of Veterinary Medicine, Universiti Putra Malaysia, Serdang 43400, Malaysia; mzamri@upm.edu.my

**Keywords:** *Theileria*, anaemia, clinical pathology, immunopathology, diagnosis, DNA vaccine

## Abstract

Theileriosis is a blood piroplasmic disease that adversely affects the livestock industry, especially in tropical and sub-tropical countries. It is caused by haemoprotozoan of the *Theileria* genus, transmitted by hard ticks and which possesses a complex life cycle. The clinical course of the disease ranges from benign to lethal, but subclinical infections can occur depending on the infecting *Theileria* species. The main clinical and clinicopathological manifestations of acute disease include fever, lymphadenopathy, anorexia and severe loss of condition, conjunctivitis, and pale mucous membranes that are associated with *Theileria*-induced immune-mediated haemolytic anaemia and/or non-regenerative anaemia. Additionally, jaundice, increases in hepatic enzymes, and variable leukocyte count changes are seen. *Theileria annulata* and *Theileria parva* induce an incomplete transformation of lymphoid and myeloid cell lineages, and these cells possess certain phenotypes of cancer cells. Pathogenic genotypes of *Theileria orientalis* have been recently associated with severe production losses in Southeast Asia and some parts of Europe. The infection and treatment method (ITM) is currently used in the control and prevention of *T. parva* infection, and recombinant vaccines are still under evaluation. The use of gene gun immunization against *T. parva* infection has been recently evaluated. This review, therefore, provides an overview of the clinicopathological and immunopathological profiles of *Theileria*-infected cattle and focus on DNA vaccines consisting of plasmid DNA with genes of interest, molecular adjuvants, and chitosan as the most promising next-generation vaccine against bovine theileriosis.

## 1. Bovine Theileriosis

Bovine theileriosis is an important tick-borne disease of domesticated cattle in tropical and subtropical countries, caused by several *Theileria* species belonging to the phylum Apicomplexa [1]. Some species cause serious economic losses through bovine mortalities and morbidities in many countries [2,3,4,5]. For example, in India, the economic loss due to blood parasite diseases in animals has been estimated to about USD 498.7 million per annum, and bovine tropical theileriosis alone caused the loss of USD 384.3 million per annum [6]. In Sub-Saharan Africa, more than one million cattle deaths from East Coast fever (the disease caused by *T. parva*) have been recorded per year, leading to the loss of about USD 300 million [7].

*Theileria* species that infect bovines include *T. annulata*, *T. parva*, *T. mutans*, *T. orientalis* complex (*orientalis/sergenti/buffeli*), *T. tarurotragi*, *T. velifera*, *T. sinensis* and *Theileria* sp. Yokoyama, a newly discovered *Theileria* species closely related to *T. annulata* [8,9,10,11,12,13]. The geographic distribution of *Theileria* species is reported in Table 1, along with the disease names they cause and the vector tick species. There is an argument regarding whether the *T. orientalis/sergenti/buffeli* group should be considered as a single or separate species individually referred. The phylogenetic analyses of *Theileria* species are based on 23S ribosomal RNA, 18S ribosomal RNA and the major piroplasm surface protein (MPSP) gene [14,15]. The sequence variations in the MPSP gene have been used to ascertain the molecular diversity of *T. orientalis* [15]. Some parasitologists support the name *T. orientalis* complex for all three benign *Theileria* species in the *T. orientalis* complex group, while others still refer to them separately, but a consensus on whether it should be considered as a single species or referred to individually is yet to be reached [16,17].

*Theileria* species possess unique characteristics that make them different from other apicomplexa such as *Toxoplasma* and *Plasmodium*, in that they do not reside inside a parasitophorous vacuole [18]. The invading *Theileria* sporozoites are free within the host cell cytosol after the rapid dissolution of the surrounding host cell membrane and immediately associate with the host cell microtubules to differentiate into schizonts [18]. Unlike other apicomplexan organisms, they are non-motile and do not have a well-developed apical complex [8]. *Theileria* spp. are transmitted by Ixodid ticks of the genera *Haemaphysalis*, *Rhipicephalus, Hyalomma* and *Amblyomma* (Table 1) [3,8]. The life cycle begins when ticks become infected after a blood meal on an infected mammalian host with parasitized red blood cells (RBCs). The ingested RBCs are lysed in the tick gut lumen, the released piroplasms transform into gametocytes and fertilization (syngamy) occurs within the gut lumen. Following fertilization, a zygote results that invades the gut epithelium, goes through meiosis, divides and transforms into motile kinetes. The kinetes migrate from the gut epithelium cells to the haemolymph, get to the salivary gland and infect a large proportion of cells. In the salivary gland, the parasite undergoes a process known as sporogony, and develops into a multinucleated sporont [19]. The infected adult ticks or nymphs slowly release thousands of infective sporozoites into the blood of the next mammalian host some days after attachment [19,20]. The sporozoites then invade the leukocytes and within a few days develop into schizonts (schizogony). At about 10 days post infection with sporozoites, schizonts can be transiently found in the lymph nodes, spleen and liver. Schizont-infected cells are seldom found in the circulating blood and are not important in the pathogenesis of benign forms of bovine theileriosis but play a major role in the pathogenesis of diseases caused by the so called schizont-transforming species [15,21]. Schizonts undergo a process of merogony and produce merozoites, which are released by host cell rupture. Merozoites invade RBCs, where they reach the piroplasm stage [19].

The schizont-transforming species include *T. annulata* and *T. parva*. The uncontrolled proliferation of the schizonts and the incomplete fatal transformation of T and B lymphocytes and monocytes [22,23] in the affected cattle are responsible for the severe course of bovine theileriosis caused by *T. annulata* and *T. parva* infections [3]. Clinical signs include fever, lethargy, enlarged lymph nodes, jaundice, anaemia, increased cardiac and respiratory rates, respiratory failure, decreased milk production, ocular discharge, abortion and still births [24,25]. Cattle that are left untreated usually die from the disease.

*Theileria annulata,* the causative agent of tropical bovine theileriosis, is regarded as the most pathogenic *Theileria* species with a worldwide distribution [10,25]. The cattle-adapted *T. parva*, which causes East Coast fever, is an economically important *Theileria* species that affects cattle in Sub-Saharan Africa. This haemoprotozoan represents a subpopulation of *T. parva* that has adapted to maintenance in cattle. Another form of the disease, known as corridor disease (CD), is caused by the infection of cattle with buffalo-derived *T. parva,* formerly known as *T. parva lawrenci* [26]. The buffalo-derived *T. parva* exhibits much greater genotypic and antigenic diversity than the cattle-adapted *T. parva* (reviewed in [27]). *Theileria parva* does not cause clinical disease in African buffalo, but infected buffalos play a major role in the epidemiology of the disease as they are natural reservoir hosts and a source of infection to *Rhipicephalus appendiculatus* ticks [28]. Buffalo-derived *T. parva* infections in cattle are characterized by a shorter course of the disease, a low number of *Theileria* schizont-infected cells, very low piroplasm parasitaemia when compared to East Coast fever [26] and buffalo-derived *T. parva* is not transmissible from infected cattle [29]. In contrast to other *Theileria* species [30], the buffalo-derived *T. parva* infection is self-limiting, with no development of a carrier state in recovered cattle [29].

The non-schizont-transforming species include emerging haemoprotozoans belonging to the *T. orientalis* complex *T. buffeli, T. sergenti,* and *T. orientalis*. Besides tick transmission [3], their transplacental transmission has been documented [30,63]. Theileriosis due to an *T. orientalis* complex exerts its major effect following the infection of the erythrocytes by the piroplasmic forms and subsequent erythrocyte destruction [21]. Recent outbreaks of oriental theileriosis in the Asia-Pacific region have shown that pathogenic genotypes of *T. orientalis* exist, which cause reduced growth and production losses in infected cattle and therefore have an economic impact [45,64,65]. Eleven genotypes (types 1-8 and N1-N3) of *T. orientalis* complex parasites have been identified based on the sequence variations in the MPSP gene, which is conserved in *T. orientalis* complex piroplasms and is expressed during the intraerythrocytic stage of the protozoa [1]. Two (*chitose* type I and *ikeda* type 2) of the 11 genotypes of *T. orientalis* have been demonstrated to be pathogenic [15,24,42,43,65] and associated with severe haematological alterations, production losses, high morbidity and mortality in affected cattle [42,65,66]. The MPSP genotype 3 is known as the *T. buffeli,* while genotype 6 was reclassified with the taxonomic name *T. sinensis* to reflect its divergence from other benign *Theileria* species [39,60]. Persistent infection of *T. orientalis* with different variants is common [24], and the immune mechanism responsible for disease resistance is not completely understood. The virulence in *T. orientalis* was considered to be genotype-related; however, one of the means by which *T. orientalis* evades the host immune system is that infection usually occurs as a set of mixed infections with more than one of its genotypes [67]. *Theileria orientalis* double or triple genotype co-infection is very common, and affected cattle seldom show any clinical signs of the disease, thereby making it difficult to diagnose subclinical and mild forms of theileriosis [65]. The outcomes of *T. orientalis* infections are highly dependent on environmental stress factors, the level of acquired immunity in the affected cattle and herd genetics [68]. Additionally, physiological factors such as pregnancy, lactation and sudden changes in temperature and humidity can lead to a relapse of clinical signs in chronically infected cattle [69]. Transient fever in conjunction with anaemia can be observed. There is usually a low level of parasitaemia in previously exposed animals [70]. Theileriosis is usually more severe when naïve animals are transported into an endemic area or when chronic carriers are brought into a herd where suitable tick species are present [24].

Information regarding the pathology associated with *T. sinensis* is very limited. Infected cattle harbor the parasite for a very long period and become sources of infection to naïve cattle [30].

## 2. Clinical Pathology of *Theileria*-Infected Cattle

### 2.1. Complete Blood Cell Count and Coagulation Profile

In *T. annulata* naturally infected cattle, marked changes in complete blood cell count (CBC) have been recorded [42,71,72]. Anaemia is a feature point in tropical bovine theileriosis and severity was positively related to parasitaemia rates [73]. *Theileria annulata* caused a regenerative anaemia with significant decreases in the RBC count, packed cell volume (PCV), haemoglobin (Hgb) concentration, mean corpuscolar haemoglobin concentration (MCHC) with increased mean corpuscular volume (MCV), and marked reticulocytosis in infected Holstein cattle [71]. In 8-day-old newborn female twin Holstein calves with fatal tropical theileriosis and high-level parasitaemia (>70%), haemolytic regenerative anaemia was severe [74]. Decreases in RBC count and Hgb concentration were recorded for Egyptian buffaloes with tropical theileriosis [75,76].

Similar to tropical theileriosis, regenerative anaemia associated with either intravascular or extravascular haemolysis [77] and significant decreases in RBC count, Hgb concentration and PCV values and an increase in MCV were observed in natural *T. orientalis* infections in cattle raised in mountainous areas [41]. In Korea, MPSP 1, 2, 3 and 7 genotypes were detected in MPSP-positive Holstein cattle with natural *T. orientalis* infection and they showed a significant more severe regenerative anaemia compared to the MPSP negatives [78]. Interestingly, anaemia was more frequent and more severe in MPSP-genotype-1-positive individuals [78]. Macrocytic normochromic anaemia was also recorded in *Theileria* species-infected Malaysian cattle [79] and in a cow with lethal *T. buffeli* infection [77].

Leukocyte count varied based on infecting *Theileria* species and strain. In fact, panleukopaenia was a remarkable finding in tropical theileriosis and Omer et al. [80] reported leukopaenia marked by lymphopaenia and thrombocytopaenia in pure-bred cattle. However, Stockham et al. [77] reported leukocytosis with marked neutrophilia, lymphocytosis and monocytosis in a beef cow with *T. buffeli* infection. Higher levels of parasitaemia in cattle with tropical theileriosis resulted in reduced lymphocyte and monocyte numbers and increased absolute neutrophil numbers [73]. Lymphopenia was reported in 8-day-old newborn female twin Holstein calves with parasitaemia >70% [74]. Significant leukocytosis with neutrophilia was observed in a cattle group with <1% parasitaemia, while a significant reduction in lymphocyte numbers and increases in neutrophil and leukocyte numbers were recorded for cattle with 1–3% parasitaemia [81]. Leukopaenia was found in Egyptian buffaloes with tropical theileriosis [75,76]. Thrombocytopaenia and panleukopaenia were remarkable findings in *T. annulata*-infected Egyptian water buffaloes [82]. Variability in reported leukocyte and platelet count changes can be due to the phase of disease. During the acute phase of the infection, there was usually an increase in the leukocyte and thrombocyte numbers, which gradually declined as the infection became chronic [79]. However, Ghanem et al. [81] reported leukopaenia in Egyptian water buffaloes with acute theileriosis. In experimental *T. parva* infection with two different strains, fluctuations in leukocyte and granulocyte counts were found, with an early decrease followed by a more severe agranulocytosis just before the animals died [83].

The coagulation profile of *T. annulata*-infected cattle includes a prolonged activated partial thromboplastin time and prothrombin time, thrombocytopenia and hyperfibrinogenaemia [71].

### 2.2. Biochemical Profile and Urinalysis

Anaemic cross-bred cows with tropical theileriosis had significant decreased levels of sodium, ionized calcium, total protein and albumin compared to non anaemic cows, and increased concentrations of blood urea nitrogen and creatinine [56]. El-Deeb and Iacob [82] reported significant increases in the levels of acute phase positive markers, such as haptoglobin, serum amyloid A, ceruloplasmin, α1-acid glycoprotein and fibrinogen levels in *T. annulata*-infected water buffaloes. Similarly, sialic acid value increased in *T. annulata*-infected Holstein cattle as well as adenosine deaminase activity, while total antioxidant capacity dropped [62].

Significant elevations in the serum activities of aspartate aminotransferase (AST), alanine aminotransferase (ALT), alkaline phosphatase (ALP), serum levels of low-density lipoprotein cholesterol (LDL), very-low-density lipoprotein cholesterol (VLDL), β-hydroxybutyrate and non-esterified free fatty acids, and significant decreases in serum albumin, globulin, cholesterol, triglyceride, glucose, calcium and phosphorus levels were notable findings in Egyptian water buffalo tropical theileriosis [76,82]. Similar findings were observed in a beef cow with lethal oriental theileriosis caused by *T. buffeli* [77].

Urinalysis was performed in 8-day-old newborn female twin Holstein calves with fatal disease and showed trace amounts of proteins in urine, haemoglobinuria and haematuria [74].

## 3. Pathophysiological Mechanisms of Anaemia in Bovine Theileriosis

### 3.1. Anaemia in Oriental Theileriosis

The pathophysiologic mechanism of anaemia in oriental theileriosis is multifarious [77]. Anaemia is the primary clinicopathological finding in oriental theileriosis and usually occurs due to intravascular haemolysis caused by the intra-erythrocytic stage. *Theileria orientalis* is also known to induce immune-mediated haemolytic anaemia [84]. The life span of erythrocytes in oriental theileriosis caused by *T. sergenti* is usually shortened as the immune system produces antibodies directed against the parasites as well as against its own erythrocytes [85,86]. Autoantibody production against RBCs is due to the altered RBC membrane, as phosphatidylserine molecules, which are normally localized on the inner leaflets of cell membranes, translocate to the external surface of RBCs in *Theileria*-infected cattle. Exposure of the phosphatidylserine on the cell surface can induce an antibody response and function as a marker of the phagocytic clearance of RBCs by macrophages [86,87]. Hagiwara et al. [88] evidenced in an experimental model with immunodeficient mice that haemolysis of *T. sergenti* infected RBCs occur without the involvement of antibodies or complement. Shiono et al. [86] demonstrated that elevations in methaemoglobin concentration contribute to the progression of anaemia, as an increase in methaemoglobin can alter the oxidant–antioxidant balance and cause oxidative damage of RBC membranes and their removal from circulation by phagocytes [69].

### 3.2. Anaemia in East Coast Fever

Mbassa et al. [36] reported, in 1994, unusual cases of East Coast fever in zebu and taurine–zebu crosses cattle in Tanzania, where the infection of the haematopoietic precursor cells resulted in severe pancytopaenia and the severe anaemia was not associated with reticulocytosis, haemoglobinuria or jaundice. Additionally, this *T. parva* strain caused lymphocytolysis in lymph nodes where lymphoproliferation was low and only few schizonts were found. Conversely, anaemia was mild and regenerative in cattle and buffaloes with East Coast fever, and numerous macrophages were present in the lymphoid organs [36]. However, non-regenerative anaemia and pancytopenia were observed in chronic forms of the disease, because *T. parva* merozoites infect erythroid and other haematopoietic precursor cells, resulting in the extensive destruction of haematopoietic cells in bone marrow [36].

## 4. Immune Response and Immunopathology in *T. parva* and *T. annulata* Infections

### 4.1. Innate and Adaptive Immune Responses in Theileria Species Infection

In bovine tropical theileriosis, the host immune response involves both the innate and adaptive branches of the immune system, which interact to protect cattle against *Theileria* infection. Innate immunity provides a front line of host defense, ensuring rapid but nonspecific defense mechanisms immediately after pathogen invasion in the host. Specific and protective immune response against all parasite stages (sporozoite, trophozoites, schizonts and merozoites) is mounted following inoculation by infected ticks [89]. In response to invading *Theileria* parasites, natural killer cells (NK), macrophages and γδ T lymphocytes secrete cytokines that modulate the interaction between immune system cells in the host. These interactions promote synergy between macrophages and NK cells and increase the interferon (IFN)-γ level, which activates macrophages and interleukin (IL)-12 for the proliferation and activation of NK cells. The innate response of macrophages and NK cells drives the adaptive response towards T helper 1 responses [90]. The adaptive immune response promotes immunity by activating macrophage microbicidal and phagocytic activities. Schizont-infected macrophages and activated uninfected macrophages secrete similar cytokines, thus suggesting that *Theileria* schizont-infected and activated uninfected macrophages have an influence on both the innate and adaptive immune response. Therefore, NK, CD8^+^ and γδ T cells produce IFN-γ, which acts on macrophages. The macrophages in turn produce nitric oxide that clears both trophozoite and schizont-infected cells. NK cells lyse schizont-infected cells, clearing schizonts while the macrophages undergo apoptosis, as reviewed in [91]. Pro-inflammatory cytokines such as tissue necrosis factor alpha (TNF-α), interleukin beta (IL-β) and interleukin 12 (IL-12) are secreted by monocytes and macrophages. These cytokines mediate the involvement of acute phase proteins and allow the T-helper cell (CD4^+^ T cells) to differentiate, thereby forming a link between innate and adaptive immunity [73]. The activated CD4^+^ T cells secrete cytokines such as IL-2 and IFN-γ, which are needed for the clonal expansion of the cytotoxic T cells [92]. Bovine macrophages are stimulated by IFN- γ to secrete TNF-α, which modulates the host defense against *Theileria* by inhibiting the trophozoite-infected cells and macro-schizont infected cells (reviewed in [91]). Nevertheless, high levels of IFN-γ have a negative effect on B cell development as they have been reported to cause a significant reduction in B cell numbers.

### 4.2. Immunopathogenesis in Cattle Tropical Theileriosis

*Theileria annulata* infections are usually fatal, especially in susceptible, imported or naïve cattle [93]. The death of affected cattle is attributed to the transformation and proliferation of schizont-infected mononuclear cells and/or haemolytic anaemia resulting from the intracellular piroplasms [94]. Significant increases in pro-inflammatory cytokines such as TNF-α, IL-1α, IL-6, IL-12, IL-Iβ and IFN-γ occur in response to the invading blood protozoa [91]. Previously, some authors have stated that the fatality due to infection is greatly dependent on the overproduction of cytokines, such as TNF-α produced by the schizont-infected monocytes/macrophages and uninfected macrophages [95,96]. The excess cytokines are responsible for pathologies associated with tropical theileriosis [93,95,96]. Tissue response in all lymphoid organs is a macrophagic hyperplasia. No transformed T cells were found in the lymphoid tissues of *T. annulata*-infected cattle when compared to the lymphoid tissue of cattle infected with *T. parva*, where an extensive population of lymphoid cells were present [93]. The immune subversion process in immunocompromised or susceptible cattle is induced by a protozaoal superantigen expressed on the schizont-infected lymphocytes. This antigen interferes with antigen recognition and presentation to cytotoxic or helper T cells [97]. In cases where *T. annulata* did not cause the destructive disease, it was shown that schizont-infected macrophages possess a powerful mechanism of immune subversion. They inhibit the proliferation of schizont-infected lymphocytes and also suppress the non-specific activation of lymphocytes induced by the superantigen-like molecule (reviewed in [91]). The dual function (the inhibition of proliferating schizont-infected cells and the suppression of non-specific lymphocyte activation) of activated macrophages explains how cattle overcome the superantigen-like activities of multiplying schizont-infected cells and succeed in controlling the infection, especially in case of immunocompetent animals (reviewed in [91]). Therefore, schizonts of *T. annulata* have the ability to convert immune cells into a rapidly expanding population of metastatic lymphocytes, and this method of immune subversion makes it distinctive among pathogens of macrophages. Furthermore, cytokines and other factors, such as nitric oxide and matrix metalloproteinases (MMP) produced by both infected and non-infected macrophages, influence the outcome of infection, suppressing the growth of parasitized cells but inducing clinical signs and causing tissue damage [91].

Flow cytometric analysis of samples from *T. annulata*-infected cattle showed a significant decrease in the T (CD4^+^ and CD8^+^) and B cell populations in case of high parasitaemia, while an increase in T and B cells was observed in cattle infected with <1% parasitaemia [73]. The decrease in circulating T cell population despite activation shows an unsuccessful response of the T cells to the secreted stimulatory cytokines (IFN-γ and TNF-α) [73,98]. Baldwin et al. [99] and Spooner et al. [100] confirmed that schizonts of *T. annulata* predominately infect macrophages, dendritic cells and B cells and are no longer regarded as protozoa that cause lymphoproliferation, while *T. parva* infects T cells, thereby causing lymphoproliferation. The different cell tropism was inferred by the type of cytokines produced by *T. annulata* schizont-infected cells, which are only characteristic of macrophages. In fact, the cytokine profile of schizont-infected monocytes/macrophages includes IFN-α, TNF-α, IL-1α, IL-1β, IL-6, and IL-10 [98]. Interestingly, the levels of T cell proliferation induced by infected cells correlated with the level of expression of the T cell stimulatory cytokines IL-1α, IL-1β, and IL-6 [98]. *T. annulata* schizonts predominately have an affinity for CD3^−^ CD11b^+^ phagocytic cells [93], which express major histocompatibility class (MHC) II antigens [97]. In vivo, *T. annulata*-infected mononuclear cells are found throughout the lymphoid organs, and their metastasis is due to their expression of MMP [101] and of adhesion molecules such as CD2, CD 11b, followed very late by the expression of antigen 4 (VL4) and CD9 [93]. When schizont-infected lymphocytes metastasize in pituitary and adrenal glands, multiple endocrinological failures can additionally occur [102].

### 4.3. Immunopathogenesis in East Coast Fever

*Theileria parva* transforms lymphocytes and inhibits cell apoptotic pathways to secure its own survival within the host cells [103]. This parasite has the capability to transform T and B lymphocytes and harmonize its own division with that of the host cell. During host cell mitosis, the schizonts attach to the mitotic spindle, ensuring that both daughter cells contain schizonts after division, thereby maintaining an enhanced and efficient infection rate [18]. Therefore, in East Coast fever, parasite multiplication is highly dependent on lymphoproliferation [22]. The features of cancer cells exhibited by *Theileria*-infected immune cells have been extensively reviewed by Tretina et al. [23]. Briefly, the transformed and immortalized *T. parva*-infected lymphocytes possess a cancer-like phenotype. However, this is a reversible process as transformed *T. parva*-infected lymphocytes return to a resting phenotype upon clearance of the parasites by the theilericidal drug buparvaquone. Therefore, the transformation of *T. parva*-infected lymphocytes is not dependent on defined genomic changes in the host cell [104]. However, a few studies have shown irreversible gene expression changes in experimentally infected *T. annulata* bovine lymphosarcoma cell lines and bovine leukaemia cells, which died from apoptosis within a couple of days [105,106]. The ability of *T. parva*-infected cells to proliferate indefinitely, avoiding apoptosis, was attributed to the suppression of p53 activity, whose primary role is to mediate host cell apoptosis but which is quickly re-activated after treatment with theilericidal drugs [107]. The p53 protein is a tumor suppressor in mammalian cells and, under normal circumstances, it is maintained at low levels due to its degradation by proteasomes and the rapid turnover [108,109]. In response to stress, p53 can accumulate and control cell cycle progression [22]. The mouse double minute 2 (MDM2) gene acts as a negative regulator of p53 by binding to the p53 NH_2_-terminus and thereby impedes its transcriptional activity [110] and can exhibit oncogenic activity when upregulated in cells. In fact, the MDM2 protein is overexpressed in about 10% of human cancers [111]. Many spliced isoforms of MDM2 are observed in humans with cancer, and the cDNA coding some of these spliced isoforms is also capable of transforming cells [22]. The overexpression and spliced isoforms of MDM2 contribute to its oncogenic function [22]. MDM2b is commonly found in numerous tumor cells [112] and was detected in *T. parva*-infected cell lines. This thereby suggested that an increase in MDM2b mRNA levels is associated with lymphocyte proliferation [22]. Hayashida et al. [22] opined that one or more of the isoforms of MDM2 were involved in the incomplete transformation of *T. parva*-infected cells through p53-independent mechanisms. The p53 protein is mainly localized within the nucleus of infected cells, but conflicting reports have shown that it is associated with the schizont membrane [107]. However, how the parasite sequesters p53 onto its schizont surface membrane is unknown. The authors suggested that the sequestration of p53 by the protozoa onto its surface membrane is a mechanism for p53 suppression in *T. parva* transformed cells, and they concluded that the active degradation of p53 by MDM2 and cytoplasmic sequestration contribute to the continuous immortalization of the host cells and the proliferation of the protozoa [22]. it is worth noting that treatment with burparvaquone led to a significant reduction or downregulation of MDM2 [22]. Additionally, anticancer agents were found to efficiently impede the proliferation of *T. parva*-infected cell lines and promote apoptosis [113]. Among them, cisplatin is an anticancer agent that resulted in p53 accumulation and induced the apoptosis of *T. parva*-infected cells [114].

Immunohistochemistry demonstrated an increased number of infiltrating IL-17 secreting CD163^+^ macrophages (alternatively activated M2 macrophages) amongst other intralesional macrophages in the pulmonary, lymphoid, splenic and hepatic tissues of *T. parva*-challenged cattle [115]. Increased numbers of infiltrating CD163^+^ macrophages in tissues have been correlated with poor prognosis [116]. *Theileria parva* schizont-infected lymphocytes produce a significant amount of IL-10 [117], which in turn upregulates CD163^+^ expression. Protozoa induce alternatively activated macrophages and trigger the anergy, exhaustion and apoptosis of T cells as a means of host immune evasion [118]. Many soluble factors produced by alternatively activated macrophages worsen the course of diseases such as human lung cancer characterized by malignant pleural effusion [116]. Alternatively activated M2 macrophages play an adverse role in experimental *Toxoplasma gondii* infection in mice [119] and in bovine protozoal infections—e.g., *Trypanosoma congolense* [120] and *T. brucei* [121] infections—by altering T cell function with consequent chronicity characterized by protozoal parasite invasion, persistence and immunosuppression in the host. The M2 macrophages express CD163^+^ haemoglobin/haptoglobin receptor protein on their cell surfaces and play a role as a haemoglobin or haptoglobin scavenger. The CD163^+^ macrophage response include an interference with CD8^+^ T cell responses and the inhibition of the proliferative activity of CD8^+^ T cells [117]. The lysis of *T. parva*-infected lymphocytes and erythrocytes leads to the release of free iron and proinflammatory conditions, resulting in necrosis and the subsequent activation of an enhanced CD163^+^ macrophage response in acute East Coast Fever [115]. It is possible that the release of iron after the destruction of *T. parva*-infected erythrocytes and lymphocytes result in increased levels of proinflammatory cytokines that activate an enhanced CD163^+^ macrophage response. Interleukin-17 is a proinflammatory cytokine that plays a role in the pathogenesis of several protozoal diseases, such as ocular toxoplasmosis [122], avian coccidiosis [123], cutaneous leishmaniasis [124], and in the inflammation of bovine mammary gland [125]. Severe cytokine-mediated pathology and tissue damage has been associated with the IL-17 response, which can be suppressed following the administration of anti-IL-17 antibodies [123]. The binding of IL-17 onto IL-17 receptors on endothelial cells activates certain pathways, such as mitogen-activated protein kinase, that leads to increased levels of cell adhesion molecules, chemokine expression and vascular inflammation [126,127]. Interleukin-17 is implicated in giant cell arteritis as it enhances endothelial cell activation, an influx of mononuclear cells and vascular damage [128]. The lymphohistiocytic vasculitis of small to medium-sized blood and lymphatic vessels is a major histopathological finding in *T. parva*-infected cattle and is a consequence of IL-17 secreting CD163^+^ macrophages.

An increased number of infiltrating macrophages is observed in the enlarged spleen and lymph nodes, the liver, and the lung of *T. parva*-challenged cattle [115]. Macrophages contain haemosiderin pigment, a feature of haematophagocytosis and increased erythrocyte turnover. The aforementioned changes are classical signs of the macrophage activation syndrome (MAS) [129], also known as haemophagocytic lymphohistiocytosis. This is an exaggerated systemic macrophage response that has been observed in several disease conditions, such as autoimmunity [130], lung cancer [116] and protozoal infections, including bovine trypanosomosis [131]. In summary, the immortalization and proliferation of *T. parva* schizont-infected lymphocytes leads to a secondary systemic macrophage activation syndrome and a lethal vasculitis [115].

## 5. Diagnosis of Bovine Theileriosis

Tentative diagnosis of theileriosis is made based on suggestive clinical signs, such as enlarged lymph nodes, pyrexia, anorexia, a loss of condition and pale mucous membranes. Confirmatory diagnosis is obtained with the microscopic examination of Giemsa-stained blood smears and lymph node fine needle aspirate smears, serological and molecular techniques. The use of the optical light microscopy method has been, in the past, the only available diagnostic tool that provided the morphological identification of blood parasites in ruminants. However, diagnosis solely based on the blood or lymph node smear method has low accuracy and is associated with technical problems [132]. The microscopic examination of thin blood smears and lymph node fine needle aspirate smears from cattle showing the acute disease are best and routinely performed to detect piroplasms in erythrocytes and macro schizonts (Koch’s blue bodies) in leukocytes, respectively [6,25]. This method is time-consuming and has low sensitivity in cases of low levels of parasitaemia or in asymptomatic carriers [133]. Thus, it is not reliable for the large-scale monitoring and screening of cattle populations. Specificity is also low, as morphologically similar blood parasites and parasites within the same genus cannot be differentiated [134]. Additionally, artefacts (e.g., stain precipitates) and Howell–Jolly bodies can be confused with intra-erythrocytic piroplasms by inexperienced microscopists.

Serological methods measure *Theileria*-specific antibodies by employing ELISA assays such as the *T. annulata* surface protein (TaSP)-ELISA [135], and the recombinant polymorphic immunodominant molecule (PIM)-ELISA [136]. The indirect fluorescent antibody technique (IFAT) has limitations due to the cross-reactivity between different *Theileria* species [137]. Mohamed et al. [135] demonstrated the high sensitivity of TaSP-ELISA when compared to the standard microscopic method and suggested its suitability for the diagnosis of *T. annulata* infection in cattle under field conditions. A recombinant antigen ELISA based on MPSP has been developed for detection of *T. orientalis* [67].

Molecular diagnostic techniques, such as PCR based on the 18S ribosomal RNA gene, MPSP gene, 28S ribosomal RNA genes and the sequencing of PCR amplicons [14,78,138] and Taqman^®^ quantitative real-time PCR (qRT-PCR) assay [139], are regarded as the most accurate because of their high sensitivity and specificity and ability to differentiate between *Theileria* species and strains. Moreover, PCR molecular techniques can detect newly emerging and mutant strains [13] and can distinguish between acute and chronic infections by the quantitation of the gene copy numbers using qRT-PCR [139]. Serological techniques and PCR were found to be more sensitive and specific than the blood or lymph node smear observation in diagnosing carrier cattle in which parasitaemia has dropped to microscopically undetectable levels [132,140] and are therefore highly recommended and utilized for epidemiological studies. Other molecular biology techniques employed for the rapid detection of *Theileria* species include the loop-mediated isothermal amplification (LAMP) assay for the detection of *T. annulata* [141], bead-based luminex xMAP technology [142] and random amplified polymorphic DNA (RAPD) [143,144]. A low-density DNA microarray kit has been designed for the detection of 12 species of tick-borne pathogens, including *Theileria* [145].

## 6. Immunization against Bovine Theileriosis and Advanced Vaccine Technology

Immunization is one of the most successful strategies for the prevention of infectious diseases and vaccines against bovine theileriosis are among the few vaccines available for protozoal diseases of animals [146].

The infection and treatment method (ITM) is currently the only immunization protocol available for *T. parva* infection [132,135]. The ITM involves the inoculation of live *T. parva* parasites, alongside the treatment with expensive depot formulation of antibiotics. The ITM is not cost-effective and has a cumbersome production process as it requires large numbers of cattle for vaccine production. It is also difficult to standardize, store and distribute [147]. Live attenuated organisms are available in some countries to prevent bovine tropical theileriosis [132].

### 6.1. Theileria Vaccines under Evaluation

The aims of an ideal vaccine is to produce the same immune protection that usually follows natural infection but without causing disease to generate long-lasting immunity, to prevent clinical disease and mortality after natural challenge and to interrupt the spread of infection to susceptible animals. Therefore, to achieve successful immunization, several factors have to be considered and they include the choice of appropriate antigen and adjuvant, dosing or immunization schedule and delivery platform. The choice of antigen is highly dependent on the ability of the antigen to express immunodominant epitopes and whether it possesses the ability to induce the production of fully neutralizing antibodies and activate cytotoxic T cell response. Adjuvants enhance the immunogenic properties of vaccines by prolonging antigen persistence, enhancing co-stimulatory signals, increasing local inflammation and stimulating lymphocytes via induced cytokines. Proinflammatory cytokines such as IL-12 and IL-2 stimulate both an innate and adaptive immune response and promote T-lymphocyte proliferation. These two cytokines could act as immunopotentiators if added to a *Theileria* subunit vaccine (Reviewed in [91]). The route of vaccine administration—e.g., intramuscular, subcutaneous, intranasal, ocular, oral or *in ovo* immunization—depends on the type of pathogen, cell tropism and the stage of infection (acute, chronic or latent). Controlled release and needle-free (transdermal/topical) approaches are new delivery methods that are still in the research and development stage.

Several vaccine trials utilizing various antigens and delivery routes have been performed against *T. parva* infections. Challenges encountered facing the production of a global *T. parva* subunit vaccine include genetic complexities of *T. parva* strains [148], the high polymorphic nature of bovine MHC loci [149], the biodiversity of *T. parva* strains [150], and the dominant cellular immune response following *T. parva* subunit vaccination [151].

In order to produce new *T. parva* vaccine antigens, Bastos et al. [147] investigated molecular and antigenic properties of Tp9 as a candidate vaccine antigen expressed by sporozoite and schizont parasite stages. They replaced a weakly functional signal peptide contained in Tp9 with a human tissue plasminogen activator signal peptide (tPA) and in this way they increased secretion of Tp9 from mammalian cells. Interestingly, they demonstrated that *T. parva*-immune cattle develop both humoral and cellular immune response to this antigen and significant amounts of IFN-γ were produced by CD4^+^ T cells following ex vivo exposure to recombinant, mammalian-expressed Tp9. Therefore, recombinant Tp9 can be further evaluated as a component of a *T. parva* subunit vaccine.

Mucosal and/or systemic antibodies—and most especially the CD8^+^ T cell response—are stimulated by antigens such as *T. parva* schizont antigens (Tp1-Tp12) [147,151,152,153], *T. parva* sporozoite p67 antigen [154] and *T. annulata* sporozoite antigen SPAG1 [155]. Specific immune responses to these antigens are required for protozoa clearance from the host. These antigens recognized by MHC class I-restricted CD8^+^ T cells have been tested for their ability to induce immune responses and have been found to be vaccine candidates. These antigens also play a role in preventing or reducing the entry of sporozoites into host lymphocytes [151]. The polymorphic immunodominant molecule (PIM) is a structurally complex protozoal protein with immunogenic properties, expressed by both sporozoite and schizont stages of *T. parva* [156], and it plays a role in sporozoite entry into lymphocytes [157]. The antigen is rich in glutamine and proline and challenged cattle mount, a powerful humoral and cellular immune response, but there is no evidence yet that it can confer or sustain long-term immunity [158]. Antigenic proteins similar to PIM—*Theileria lestoquardi* surface protein (TlSP) and *Theileria annulata* surface protein (TaSP)—are expressed in *T. lestoquardi* [159] and *T. annulata* [160], respectively. Both have been demonstrated as possible components of a subunit vaccine [160]. The development of a subunit vaccine against one parasite species can protect against the other. Nene and Morrison [161] extensively reviewed several approaches to vaccination against *T. parva* and *T. annulata* and suggested that a *T. annulata* subunit vaccine is likely to protect against *T. parva* infections. This is because p67 and SPAG1 antigens can confer cross-species immunity [162], even though their protein sequence similarity is only 47% [163]. The ability to confer cross-immunity was attributed to the highly conserved epitope sequences between them, meaning that anti-p67 serum recognizes SPAG1 and neutralizes *T. annulata* sporozoites, and vice versa.

The development of an effective subunit vaccine or live vaccine against *T. orientalis* complex may not be feasible, although the development of a live vaccine based on one or two of the *T. orientalis* benign genotypes may be considered. Even though a certain immunological and genetic diversity exists among them, they are clustered together on one clade of the phylogenetic tree. It was proposed that, since they are grouped together, a cross-immunity between genotypes may exist [164]. The use of variable piroplasm surface proteins to develop a subunit vaccine against *T. orientalis* has had no success to date, and progress for this approach has been negligible over the years. Globally, there is no suitable vaccine against *T. orientalis* complex infection in cattle to date [164,165]. This is because of the difficulty of extracting pure isolates for studies, as the benign form of the disease is caused by more than one genotype of *T. orientalis* and there is a low parasitaemia [166]. The *buffeli/chitose* genotypes are more closely related to each other than to the *ikeda* genotype; therefore, this genetic diversity may have implications for vaccine design [167]. It is not yet certain if the *buffeli/chitose* genotypes can stimulate heterologous immunity against the *ikeda* genotype. This lack of suitable vaccines further complicates *T. orientalis* management. Despite the development of a live vaccine being much more feasible than the subunit vaccine, Jenkins and Bogema [67] opined that MPSP still represents a promising target for a subunit vaccine against *T. orientalis* complex.

### 6.2. DNA Vaccines

A possible solution to challenges facing the use of a *T. parva* subunit vaccine is next-generation vaccine technology based on DNA vaccines. A DNA vaccine is particularly attractive for the prophylaxis of intracellular pathogens such as herpes simplex virus and mycobacteria, and since the *Theileria* parasites are intracellular pathogens, a DNA vaccine that expresses cytokines should be appropriate [168,169]. In fact, DNA vaccines have a strong capacity to induce cell-mediated immune responses characterized by the production of T helper 1 cytokines (IL-12, IFN-γ, TNF-α, IL-21). These cytokines are a critical component in the host defense against chronic/persistent pathogenic infections and facilitate the adjuvant activity of DNA-based vaccines [170]. For instance, IL-21 can be used as a molecular adjuvant because of its involvement in T cell and NK cell activation, and its effect on CD4^+^ T cells can aid in the response to chronic or latent infection. An increased IFN-γ level enhances the activities of cytotoxic T lymphocytes and NK cells. The use of DNA constructs encoding molecular adjuvants such as IL-6 and TNF-α has proven useful in several cattle diseases, such as foot and mouth disease. The addition of a molecular adjuvant enhanced antigen-specific cell mediated responses elicited by the DNA vaccine [171]. DNA vaccination with chitosan nanoparticles has also been used in vaccination against *Staphylococcus aureus* in dairy cows [172]. Recombinant DNA vaccine constructs encoding the Tp1, Tp2, Tp4, Tp5 and Tp8 antigens have been previously described [152]. The adjuvant activity of DNA constructs expressing bovine foetal liver tyrosine kinase 3 ligand (Flt3L) and granulocyte macrophage-colony stimulating factor (GM-CSF) has been proven in vivo by Mwangi et al. [173]. These cytokines induced the recruitment of an increased number of dendritic cells to the site of inoculation and enhanced antigen-specific CD4^+^ T cell responses [173]. However, the administration of the bovine Flt3L and GM-CSF vaccine prior to DNA vaccination in *T. parva*-challenged cattle induced CD4^+^ and CD8^+^ T cell IFN-y responses but not the antigen-specific cytotoxic T -ymphocyte (CTL) response [174].

By delivering DNA via different routes, DNA vaccines can generate a different type of immune response (cellular and/or humoral). For instance, intradermal injection of DNA vaccine elicits a predominate Th1 response, while the so called biological ballistic or biolistic DNA injections mainly stimulate a Th2 or a balanced Th1/Th2 response [175]. Fry et al. [176] demonstrated for the first time the particle-mediated epidermal delivery of a DNA vaccine whereby a DNA-encoded *T. parva* codon-optimized and native sequence PIM antigen was delivered through the intra-dermal route in Holstein steers. This method is also known as gene gun immunization, whereby DNA-encoded antigens are delivered directly into the nucleus of epidermal and dermal professional and non-professional antigen-presenting cells. These antigens are then expressed and processed to elicit an immune response. The gene gun has been used for the delivery of influenza vaccine in ferrets [177]. The advantage that gene gun immunization has over traditional intramuscular DNA immunization is that it requires 10 to 100-fold less DNA and yet elicits a strong humoral and cellular immune response [178]. The use of gene gun immunization against *T. parva* infection elicited a robust protective immune response characterized by significant antibody and cell-mediated responses (Th1/IgG2 and INF-γ responses). Although the antibody response mounted was not enough to prevent East Coast fever in the *T. parva*-challenged calves, gene gun immunization may serve in the future as a suitable vaccine platform against *T. parva* and other bovine blood pathogens in cattle that sufficiently express the MHC class I molecules, with the role of binding and presenting PIM epitopes to cytotoxic T cells [176]. 

## 7. Conclusions

*Theileria* species-infected cattle remain carriers for life and serve as a source of infection to naïve cattle thanks to the wide distribution of tick vectors and suitable tick habitats. Therefore, an effective vaccine able to prevent at least losses caused by acute clinical diseases from the most pathogenic *Theileria* species would have a significant economic impact. We propose a further investigation into DNA vaccine technology for the control and prevention of bovine theileriosis, since this technology is potent against latent/persistent or chronic infections and is characterized by sustained cellular and humoral immune responses. The epidermal injection of plasmid DNA encoding *Theileria* antigens generates both humoral and cellular immune response against invading protozoa. Moreover, DNA therapeutic vaccines can be delivered repeatedly without initiating an immune response against the DNA plasmid. Importantly, they are simple and cost-effective to produce in large quantities and are more heat stable than conventional vaccines. Their temperature stability is a great advantage for use, particularly in countries where cold chain maintenance is problematic.

Co-immunization with immune-modulatory adjuvants such as plasmids encoding specific cytokines and chemokines as part of the vaccine formulation boosts the adaptive immunity. Chitosan—an easily absorbed, biodegradable nanoparticle—should be considered as a suitable antigen carrier as it permits slow release and sustains gene expression. The administration of plasmid DNA-encoding immunogenic proteins of a pathogen stands out as a novel approach for developing new-generation vaccines for the prevention of bovine theileriosis.

Currently, research is continuing into the exploration of the optimal route of DNA vaccination, the best antigen candidates, the dose and dosing/immunisation schedule for *Theileria* vaccines. However, the complexity of immunization against theileriosis emerged and, in the future, an integrated approach for effective bovine theileriosis control and eradication programs could combine the administration of next-generation vaccines and theilericidal drugs and the genetic selection of *Theileria*-tolerant or resistant cattle.

## Figures and Tables

**Table 1:** Geographical distribution of *Theileria* species infecting bovine and their ixodid tick vectors. The names of diseases caused by pathogenic species are reported in brackets.

*Theileria* Species (Disease Names)	Ixodid Vectors	Geographic Distribution	References
*Theileria annulata* (tropical theileriosis/mediterranean theileriosis)	*Hyalomma* species	Asia (India, China), Africa (Egypt, Sudan), Middle East, Europe (Portugal)	[10,31,32,33,34,35]
*Theileria parva* (East Coast fever)*,* buffalo-derived *T. parva* (Corridor Disease)	*Rhipicephalus appendiculatus, R. duttoni, R. appendiculatus, R. zambesiensis*	Africa (Kenya, Tanzania, Uganda, Mozambique)	[2,26,36,37,38]
*Theileria orientalis* (*orientalis/sergenti/buffeli*) (Oriental theileriosis)	*Haemaphysalis longicornis; Rhipicephalus microplus*	Oceania region (Australia, New Zealand), Europe (Russia, Greece, Italy, Spain, Portugal, Hungary), Asia (Vietnam, China, Korea, Malaysia, India), Africa (Ethiopia)	[24,39,40,41,42,43,44,45,46,47,48,49,50,51,52]
*Theileria taurotragi* (Benign African theileriosis)	*Rhipicephalus appendiculatus, R. zambeziensis, R. pulchellus*	Africa (Kenya, South Africa, Zambia)	[2,53,54]
*Theileria mutans* (Benign theileriosis)	*Amblyomma* species	Africa (Kenya, Ethiopia, South Africa), Central America (Caribbean Islands)	[2,55,56,57,58,59]
*Theileria velifera* (Benign theileriosis)	*Amblyomma variegatum*	Africa (Kenya, Ethiopia, South Africa), Central America (Caribbean Islands)	[2,56,58,59]
*Theileria sinensis* (Benign theileriosis)	*Haemaphysalis qinghaiensis; H. bispinosa; Rhipicephalus microplus;*	Asia (China, Malaysia), Europe (Russia)	[3,60,61,62]
*Theileria* sp. Yokoyama	Not known	Asia (Sri Lanka)	[13]
